# An Unusual Inverted Saline Microbial Mat Community in an Interdune Sabkha in the Rub' al Khali (the Empty Quarter), United Arab Emirates

**DOI:** 10.1371/journal.pone.0150342

**Published:** 2016-03-16

**Authors:** Christopher P. McKay, Jon C. Rask, Angela M. Detweiler, Brad M. Bebout, R. Craig Everroad, Jackson Z. Lee, Jeffrey P. Chanton, Marisa H. Mayer, Adrian A. L. Caraballo, Bennett Kapili, Meshgan Al-Awar, Asma Al-Farraj

**Affiliations:** 1 NASA Ames Research Center, Moffett Field, California, United States of America; 2 Bay Area Environmental Research Institute, Petaluma, California, United States of America; 3 Department of Earth, Ocean and Atmospheric Science, Florida State University, Tallahassee, Florida, United States of America; 4 Research and Studies Center, Dubai Police Academy, Dubai, United Arab Emirates; 5 Geography Department, United Arab Emirates University, Al Ain, United Arab Emirates; Auckland University of Technology, NEW ZEALAND

## Abstract

Salt flats (sabkha) are a recognized habitat for microbial life in desert environments and as analogs of habitats for possible life on Mars. Here we report on the physical setting and microbiology of interdune sabkhas among the large dunes in the Rub' al Khali (the Empty Quarter) in Liwa Oasis, United Arab Emirates. The salt flats, composed of gypsum and halite, are moistened by relatively fresh ground water. The result is a salinity gradient that is inverted compared to most salt flat communities with the hypersaline layer at the top and freshwater layers below. We describe and characterize a rich photosynthetically-based microbial ecosystem that is protected from the arid outside environment by a translucent salt crust. Gases collected from sediments under shallow ponds in the sabkha contain methane in concentrations as high as 3400 ppm. The salt crust could preserve biomarkers and other evidence for life in the salt after it dries out. Chloride-filled depressions have been identified on Mars and although surface flow of water is unlikely on Mars today, ground water is possible. Such a near surface system with modern groundwater flowing under ancient salt deposits could be present on Mars and could be accessed by surface rovers.

## Introduction

Hypersaline environments are often found in deserts where intense evaporation and low levels of water input create concentrations of salt. Such environments are of interest as examples of life in extremes and are also relevant to the question of life on Mars–a cold desert world. Salt flats are relevant analogs for habitats for life on Mars both because salt can stabilize water as liquid at low temperature and pressure and because a salt crust can preserve evidence of past life. Accordingly, there has been considerable discussion of the relevance of coastal salt flats of marine origin as possible environments for life on Mars and as sites of preservation of biomarkers [1–4]. Desert salt flats have been studied as well. Davila et al. [5] reported on hydroscopic salts trapping atmospheric water to supply photosynthetic microbes in salt domes in the driest regions of the Atacama Desert [6]. Douglas and coworkers [7–9] conducted a series of studies in the mineralogical and microbiological properties of the Badwater salt flat in Death Valley, California. Barbieri et al. [10] considered organic preservation and biosignatures in dry salt flats of gypsum from Upper Pleistocene evaporite deposits of the Chott el Gharsa, a wide continental “sabkha” (a transliteration of the Arabic word for a salt flat) in southern Tunisia. Coastal sabkha are present along the Arabian Gulf (a.k.a. the Persian Gulf) along the northern coastline of the United Arab Emirates (UAE) [11,12] as subtidal flats, intertidal flats, and in particular as extensive supratidal flats [13]. There are also sabkhas deep inland in the UAE in the Liwa Oasis within the dunes of the Rub' al Khali (the Empty Quarter) which is an extensive area of aeolian dunes covering much of the Arabian Peninsula, including part of the UAE and eastern Saudi Arabia [14]. Small basins between the dunes connect to the water table, creating a flat level below which aeolian deflation cannot readily occur [14]. The interdune flats may be sites of erosion or deposition, and may be classified according to moisture content as dry, damp, wet, or evaporitic [14] and can contain salt crusts [14–16]. These inland sabkha are an interesting analog for salt deposits on Mars and are the focus of this study.

Salt deposits are important targets in the search for life on Mars. Recent orbital data have indicated brine deposits associated with the seasonal dark streaks on Mars known as recurring slope lineae [17]. The indicated salts are calcium perchlorate and magnesium perchlorate. However chloride salts are expected on Mars as well. Orbital spectral data indicates that there are chloride-bearing deposits, likely formed in an evaporitic environment in the ancient geologic regions of Mars [18–20]. The surface conditions at these locations are currently too dry to support life and the pressure is too low to allow liquid water. The deposits on Mars are at the low levels in the basin but the elevation of the deposits is high, 1260 m above the average Martian surface [18]. The inland sabkhas of the Liwa Oasis may be an analog for how these Martian salt flats could support life just below the surface in the present climate of Mars.

**Fig 1:**
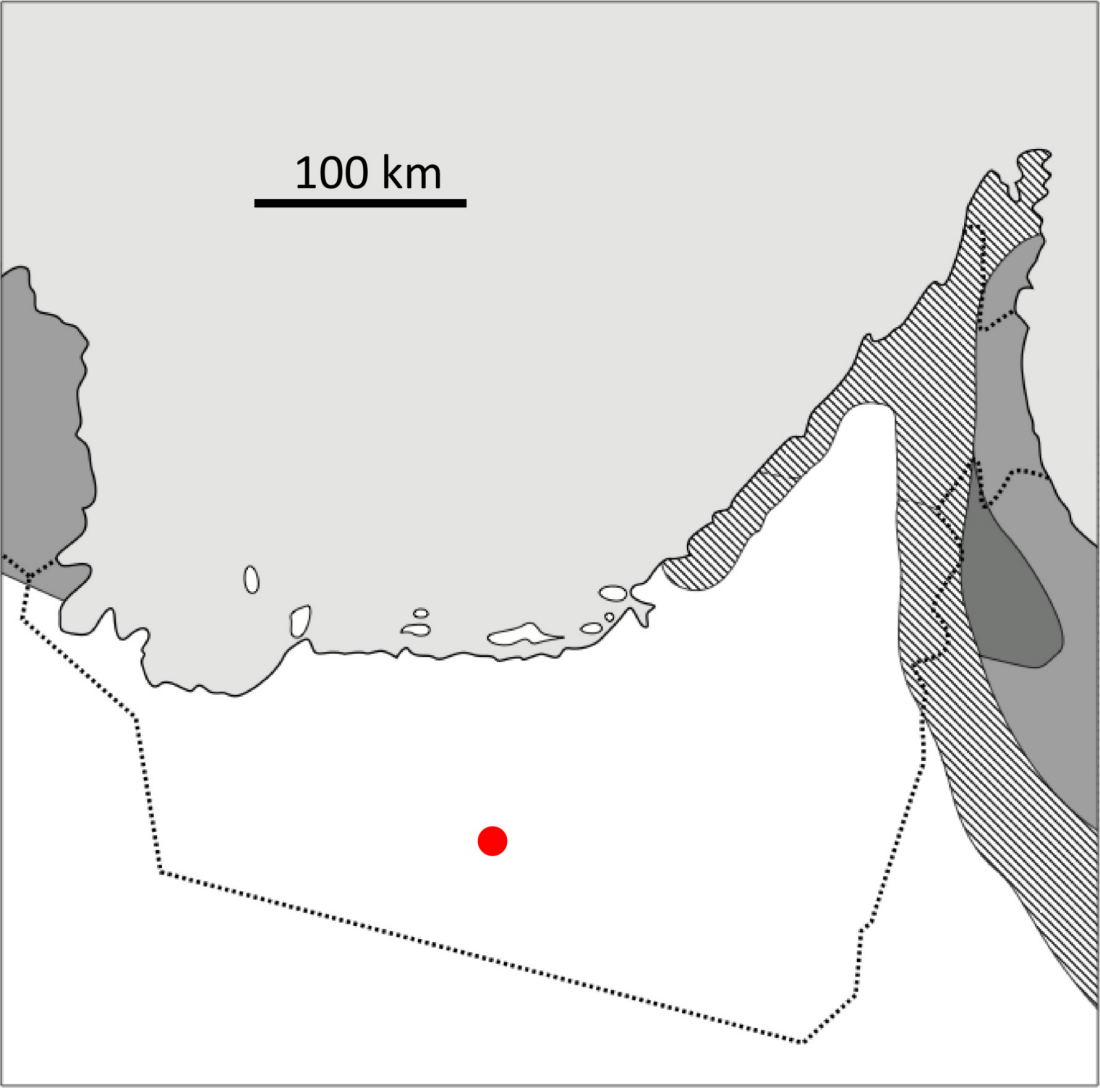
Map adapted from Böer [27] showing the approximate field location (red dot) of the Liwa Oasis Sabkhas. White areas are hyper-arid, stippled grey areas are arid, dark areas are semi-arid and grey areas are sub-humid. Very light grey is the Arabian Gulf. The UAE boader is shown as a dotted line.

**Fig 2:**
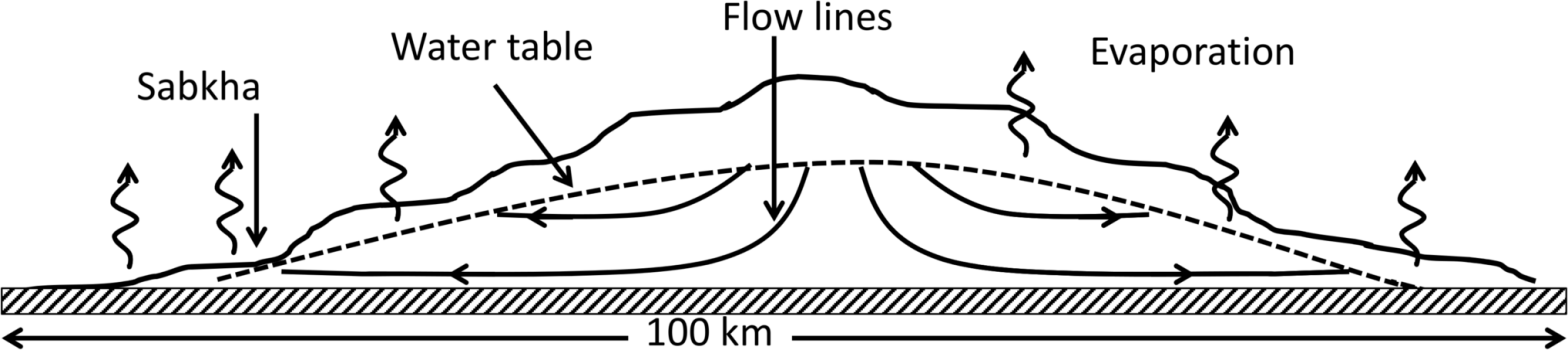
Schematic of the aquifer contained within the dunes of the Liwa Oasis. The base of the dunes is a relatively impermeable layer of carbonate close to sea level (striped layer). The solid line represents the dune profile, the dotted line the aquifer. Flow lines are shown as solid arrows and evaporation is indicated as wavy arrows. Discharge occurs at the lower elevations of the dune where the water table comes close to or intersects the surface. Evaporation occurs leaving behind salt. In the flat interdune spaces salt flats (sabkha) are formed. Figure adapted from Fig 2 of Wood and Imes [51].

The hydrology of the Liwa Oasis has been well-studied because the shallow aquifer is an important source of fresh water for human use. Thus, the age and structure of the aquifer, the flow and composition of the groundwater, and the nature of the salt flats, are well-described [16,21–26] and summarized briefly here. The location of the Liwa Oasis is shown in Fig 1 [27]. The landscape is dominated by large sand dunes with interdune spaces [14,15,28]. The sand is deposited on a flat layer of middle Miocene-age carbonates, which is essentially at sea level [16]. The aquifer contains water that is held in the sand dunes bounded below by the impermeable carbonate layer [16]. This is shown schematically in Fig 2. The water table crests in the interior of the dune field and slopes downward following the topography of the dunes. In the interdune areas, particularly near the edge of the dune field, the level of the ground water can reach the surface and wet surfaces are produced. Over time these wet areas accumulate solutes from evaporation and a salt flat (sabkha) is formed.

Radiocarbon dating and models of the groundwater formation for the Liwa Oasis [16] imply that the groundwater formed when the rainfall was approximately 20 ± 5 cm yr^-1^ between 6,200 and 9,000 yr before present and that the shift from wet to dry took place relatively rapidly between 5,500 and 6,200 yr ago. The 1990 predevelopment elevations of this water table are reported at 105 m for the crest and 85 m for the locations of discharge near the edges of the sand dunes [16]. The composition of the salts in the ground water is dominated by CaSO_4_ and NaCl [16]. Measurements of the Cl content of the groundwater near the crest of the aquifer [16] indicated levels ~1–2% of seawater. Human consumption of this fresh water source is exceeding recharge and lowering the groundwater table [16].

The structure of the Liwa Oasis interdune salt flats are inverted compared to most salt flats. In most locations with layers of saturated salt a continuing inflow from rain or tides brings a surface flow of relatively low salinity water, typically fresh water or seawater, onto the top of the salt-saturated layers. Thus, the salinity generally increases downward from either fresh or seawater at the surface to saturated salt at the bottom. The Liwa sabkha are unusual in that the surface is a thin layer of saturated salt and the relatively fresh water is flowing in from below the surface. The ground water level throughout the area of the sabkha is at, or just below, the surface. The future lowering of the level of the fresh ground water will alter the nature of the interdune sabkha communities. At the present time, the ground water reaches the surface of the sabhka site we studied and thus, just a few tens of cm below the salt flat surface, is relatively fresh water. This pattern is due to the thin translucent salt crust surface, the upward flow of fresh water at the site, and the lack of surface water flow in the desert environment.

In this paper we report on studies of the physical and microbiological composition of the inverted salt flat community in a Liwa Oasis sabkha. The interaction between the salt flat and the ground water provides a sheltered habitat for microbial life in the desert. We document the spatial distribution and identity of the organisms that live in this salinity and light gradient based on 16S ribosomal RNA (rRNA) gene sequences as well as the sequences of the *mcrA* (methyl coenzyme-M reductase) gene, which is present only in methane-producing microorganisms. Microbial communities of other arid environments, predominantly hypersaline microbial mats, are known to contain methanogens, and to produce methane [4,29–31]. This is of interest both because methane is an important greenhouse gas in Earth’s atmosphere and because of the possible *in situ* detection of Martian methane by the Curiosity rover on Mars [32].

## Materials and Methods

### Study site

**Fig 3:**
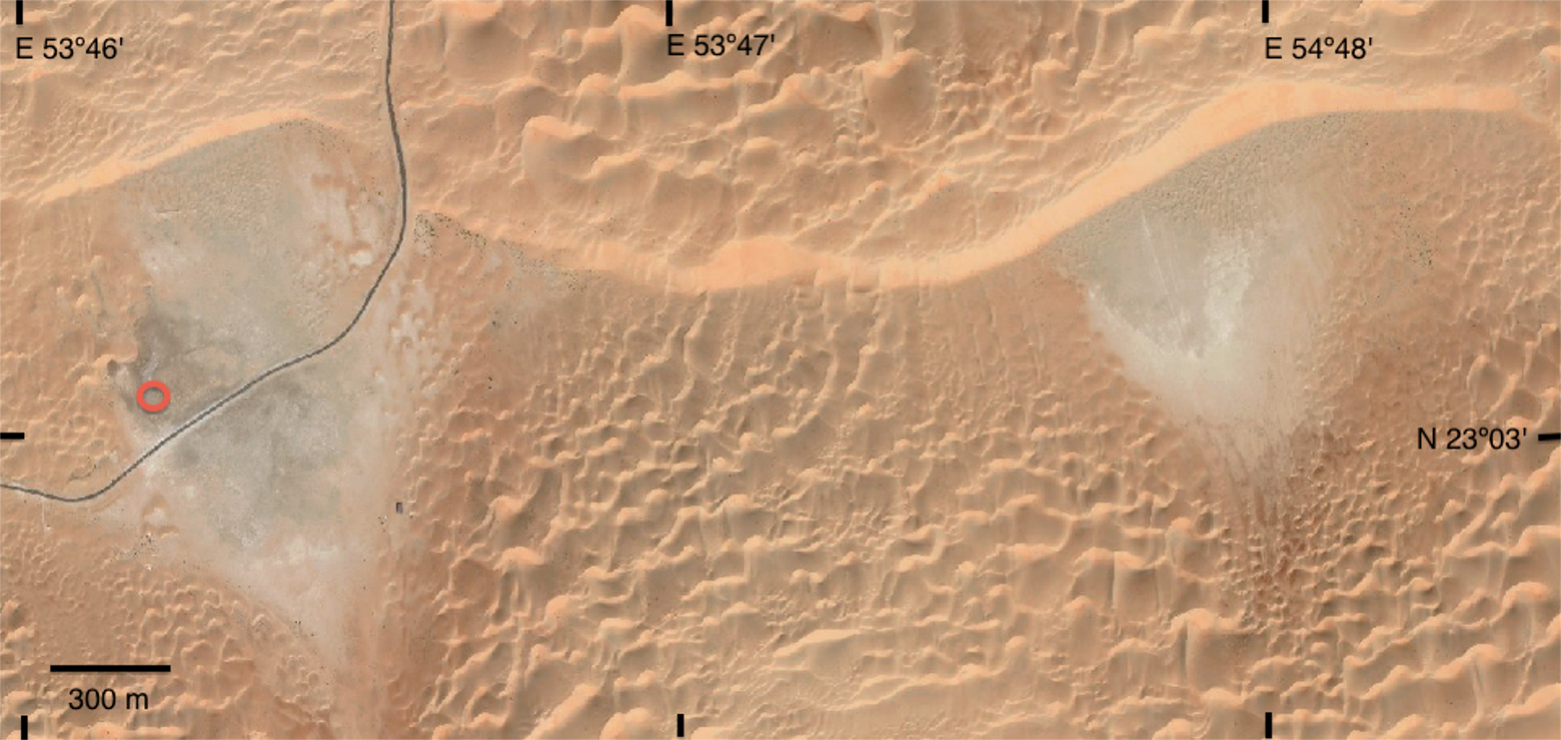
Satellite image of the inland sabkha studied here (dark area in the left side of image). The red circle marks the sampling site, GPS coordinates of the sampling site in the center of the sabkha are N23.05088° E53.77005° elevation 86 m. Another interdune flat area is visible about 1 km to the east. North is upward. Image was taken on 4 Jan 2015. Image from Digital Globe to NASA with no restrictions on use or copying.

The field studies did not involve endangered or protected species. A permit to conduct research in the United Arab Emirates was obtained through the University of UAE via our co-author A.A. Fig 1 shows the location of the Liwa Oasis and the extent of the hyper-arid zone in southwestern UAE. The study site is located at N23.05088° E053.77005° at an elevation of 86 m. Fig 3 shows a satellite image of the sabkha investigated here (dark area in the left part of figure) and another nearby interdune flat area east of the study site. Interestingly, the surface of the interdune flat area in the eastern site was not buried with sand in earlier online images (2009–2010) but was buried on the date of our field work (February 2011) and is buried in images from 2014 onward as well.

**Fig 4:**
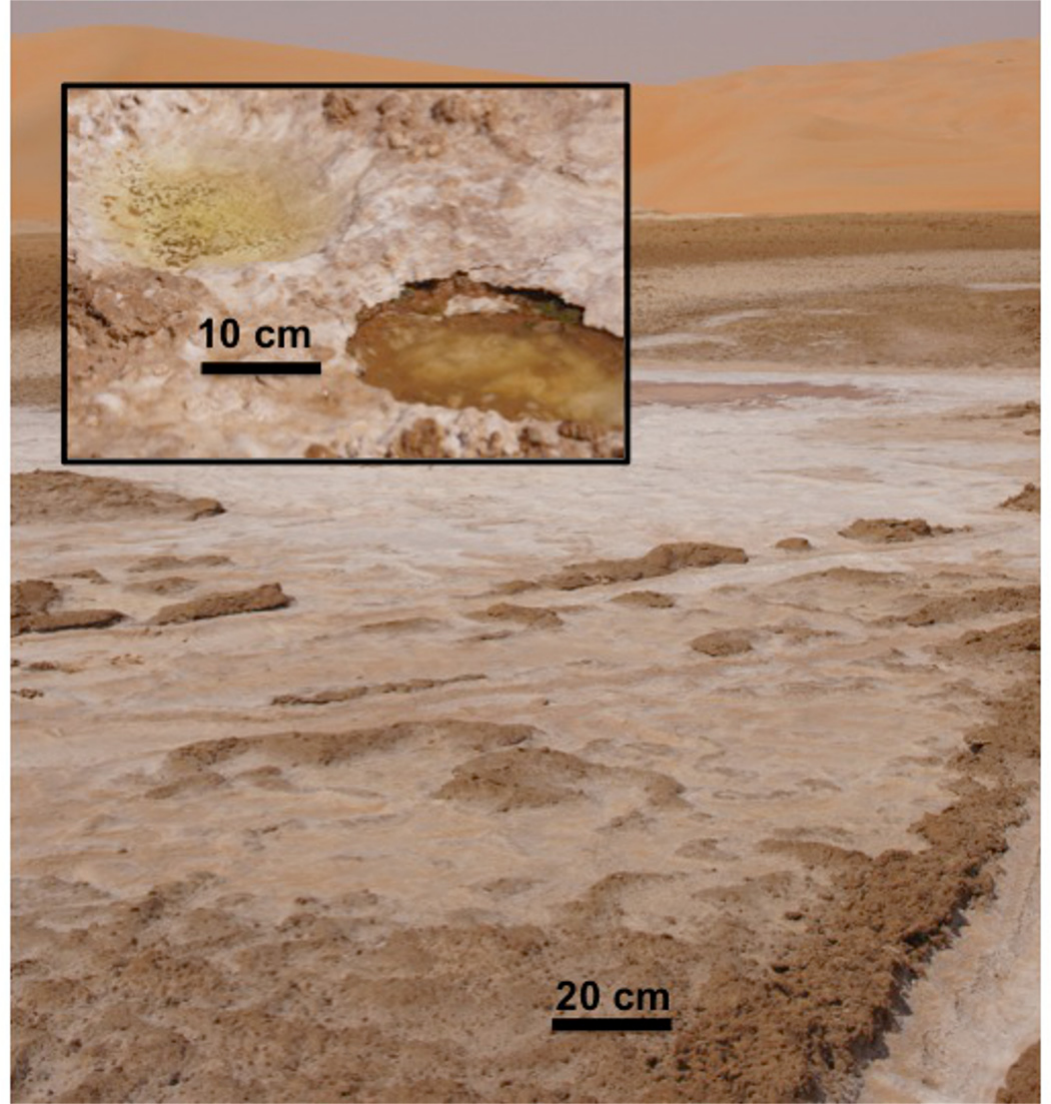
Perspective image of the salt flat surface (background image) with insert of a sampling indentation made to access the water and sediments.

**Fig 5:**
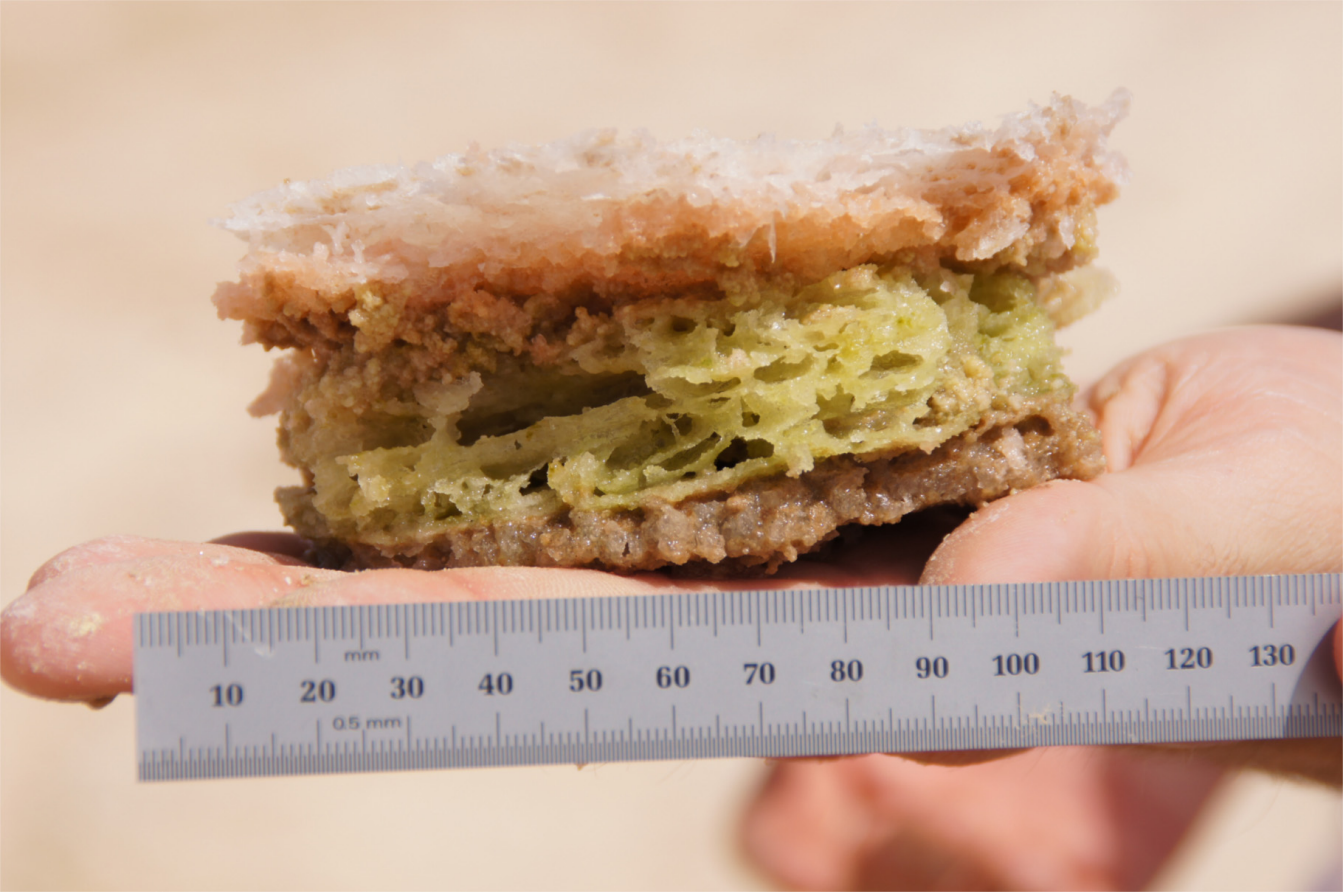
Close up of an endoevaporite mat. The uppermost white layer, about 5 mm thick, is the salt crust. Below that is a layer, almost 5 mm thick of pink halophilic bacteria and below that a layer of green photosynthetic organisms. Below the green layer the material is darker in color. Gas samples were acquired from sediments beneath this mat. The mat pictured above was sectioned from top to bottom as follows–top layer (T) composed of white and pink layer; middle layer (M) composed of green layer; bottom layer (B) composed of darker brown layer; sediment layer (S) mostly composed of fine sand attached to the bottom of the dark brown layer.

**Fig 6:**
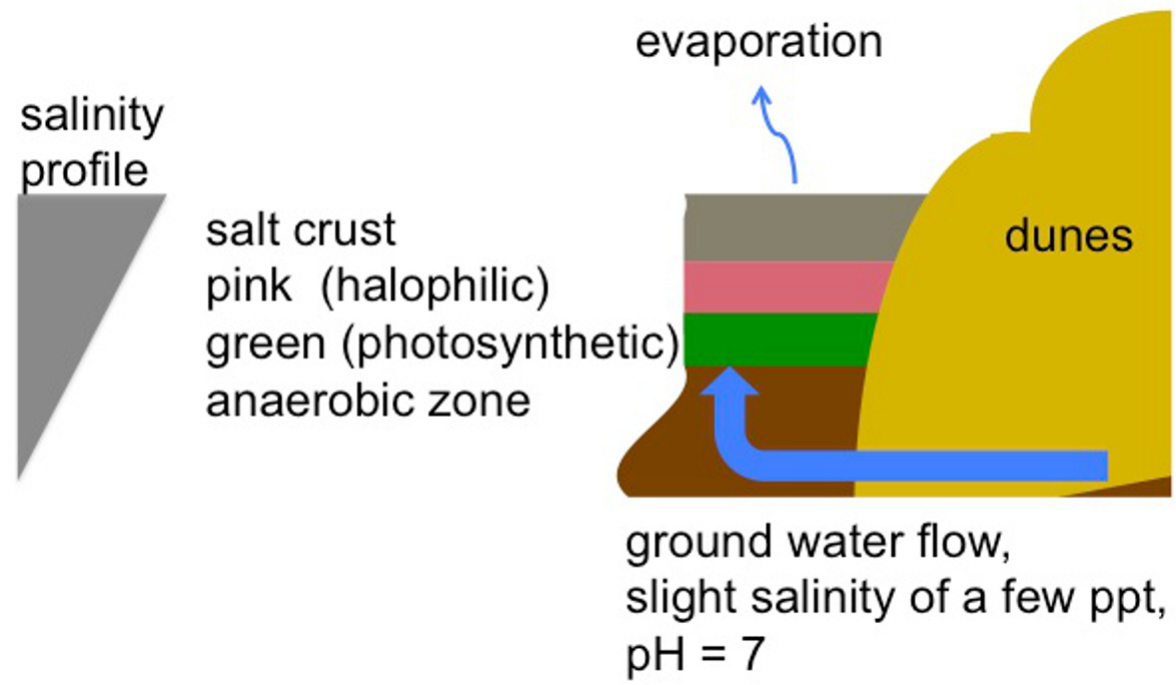
Diagram of the water flow and salt layers of the Liwa inland sabkha. Relatively fresh ground water flows upward into overtopping ancient salt layer. The salt layer inhibits evaporation, increases the salinity of the water near the surface, and allows some sunlight penetration.

At the study site there is a salt crust on the surface. This is seen in the foreground image in Fig 4. The crust is compacted and can easily support pedestrian and vehicular traffic. Our sample of the crust/endoevaporitic mat is shown in Fig 5. Breaking the crust reveals wet sediments below the surface crust layer. This is shown in the insert image in Fig 4 and the overall vertical structure of the system is shown schematically in Fig 6. Endoevaporitic mats, water and gas samples were gathered at two nearby locations in the sabkha.

### Biological samples

Endoevaporitic mats were gathered by breaking away pieces from the overlying crust (as seen in the insert in Fig 4). The mat shown in Fig 5 was brought back moist to NASA Ames Research Center and sectioned at different depths based on the colored layers and then frozen at -80°C for later extraction and sequencing to characterize the bacterial community in each layer.

### Water samples

Water collected from the two sampling pools (Fig 4 insert) was measured for salinity and pH. Gases collected as bubbles were analyzed for O_2_, N_2_, CO_2_ and CH_4_ content and ^13^C/^12^C isotope values. Water samples were collected from underneath the crust into sterile 50 mL conical tubes. pH was measured on site using a photometer (Lovibond, CheckDirect, UK) and phenol red tablets. Salinity was measured using a refractometer.

### Gas samples

Gas samples were collected from the same volume of material as the water samples, down to 20 cm, below the mats in the shallow sabkha water pools by prodding with a metal rod. Gas bubbles produced by this disturbance were captured by water displacement in an inverted funnel fitted with a syringe seal held over the area of sediments being disturbed. A syringe needle was inserted through the seal, and the captured gas in the funnel was removed and re-injected into evacuated serum bottles. Three gas samples were collected at each of the two shallow pools.

Methane (CH_4_), carbon dioxide (CO_2_), oxygen (O_2_) and nitrogen (N_2_) concentrations were determined at NASA Ames Research Center using a Shimadzu 14A gas chromatograph (GC). Methane concentrations were measured with a flame ionization detector fitted with a Porapak N column, with N_2_ as the carrier gas. CO_2_ was measured using a thermal conductivity detector (TCD) fitted with a Porapak N column with helium as the carrier gas. N_2_ and O_2_ were analyzed using a TCD fitted with a molecular sieve packed column, with helium as the carrier gas. Matheson Tri-gas 100 ppm CH_4_, 20% O_2_/79% N_2_, and 1% CO_2_ standards were run along with samples.

Gas samples for stable carbon isotopic analysis of methane were processed at Florida State University. Methane concentrations were determined using a Shimadzu Mini-2 gas chromatograph (GC) with a flame-ionization detector, fitted with a 1.83-m, 1/8in. (3.1 mm) stainless steel tubing packed with HayeSep Q 80/100 mesh. All samples were handled by directly injecting the gas (~250 μL) into the GC, where the sample was carried by UHP helium for analysis. Multiple methane standards (Scott Gas, PA, USA) were run along with samples. The analytical errors for methane concentration analyses are ± 0.5 ppm. Stable carbon isotope values were obtained using a modified cryofocusing method to amplify the methane peak [4,33]. Isotope analyses were performed using the methods described in detail in Tazaz et al. [4] and are only briefly summarized here. Duplicate analyses were performed on all gas samples. Carbon isotope data are reported in the ‘‘del” notation (e.g., δ^13^C) [4]:

$$\delta^{13}\text{C}=1000\left[{}^{13}{\text{C}/}^{12}{\text{C sam}/}^{13}{\text{C}/}^{12}\text{C std}–1\right],$$

where sam refers to the sample and std to the standard, Pee Dee belemnite (PDB). The units of δ are permil (‰). The analytical errors for stable isotopic analyses are ± 0.4‰ for δ^13^C in methane.

### Nucleic acid extractions

Genomic DNA was extracted from each layer of the mat, hereafter referred as top, middle, bottom and sediment layers (Fig 5), in triplicate using the MO BIO Power Biofilm DNA isolation kit (#24000–50; MO BIO Laboratories, Inc., Carlsbad, CA, USA) and a bead beater to lyse the cells according to the product manual. The extracted DNA was analyzed on an agarose gel to assess the quality of the extractions, and quantified on a spectrophotometer (P330 nanophotometer; Implen Inc., Westlake Village, CA, USA).

### PCR amplification

Bacterial 16S rRNA genes for all DNA extractions were amplified using the universal primers 8F (5’ -AGAGTTTGATCCTGGCTCAG -3’) and 1492R (5’ -GGTTACCTTGTTACGACTT- 3’) [34,35]. Triplicate PCR reactions were run for each sample, with each PCR mixture containing 0.6 μL of each primer (50 μM), 12.5 μL of GoTaq Green master mix (M7122; Promega, Madison, WI, USA), 1 μL of DNA template (~20 ng μL^-1^) and 10.3 μL of nuclease-free water for a maximum reaction volume of 25 μL. The 16S rRNA PCR program was set to: 95°C for 10 min, followed by 28 cycles each at 95°C for 0.5 min, 55°C for 1 min, 72°C for 1.5 min, ending with one final extension cycle at 72°C for 12 min in a Peltier thermocycler PTC-200 (MJ Research Inc., Waltham, MA, USA). The *mcrA* gene was amplified using the *mcrA* primers described in Luton et al. [36]. Triplicate PCR reactions were run for each sample, with each PCR mixture containing 1 μL of each primer (10 μM), 12.5 μL of GoTaq Green master mix (M7122; Promega, Madison, WI, USA), 1 μL of DNA template (~20 ng μL^-1^) and 9.5 μL of nuclease-free water for a maximum reaction volume of 25 μL, using the following PCR program: 95°C for 1 min, 35 cycles at 94°C for 30 s, 55°C for 30 s, 72°C for 1 min with a final extension cycle at 72°C for 5 min according to Garcia-Maldonado et al. 2014 [37]. A second amplification was performed for 25 cycles using 2 μL of *mcrA* PCR product from the first amplification to get more distinct bands. All amplifications were analyzed along with positive and negative controls for quality control.

Replicate PCR products were pooled and run on an agarose gel for each gene. The 16S rRNA and *mcrA* fragments were excised and purified using the Omega E.Z.N.A. gel extraction kit (D2500-01; Omega Bio-Tek Inc., Norcross, GA, USA) according to manufacturer instructions. Prior to cloning, a poly(A) tail reaction was performed on all 16S rRNA samples by adding 6 μL of PCR product, 1 μL 10X PCR buffer, 1 μL MgCl_2_, 1 μL dNTP, and 1 μL Taq polymerase to a total volume of 10 μL per sample and heating the samples to 72°C for 20 min in a thermo cycler.

### Cloning

The Life Technologies TOPO TA Cloning Kit for Sequencing (#K457501) (Life Technologies, Carlsbad, CA, USA) was used to ligate 4 μL of the purified PCR product to 1 μL of cloning vector (pCR4-TOPO) in addition to 1 μL of salt solution, followed by the vector-PCR insertion into One Shot Top10 Chemically Competent *E*. *coli* cells according to the product manual. The cells were plated at different densities on Luria Broth (LB) agar plates containing 50 μg μL^-1^ of ampicillin, 0.2 mM IPTG and 40 μg mL^-1^ X-gal, and were incubated at 37°C overnight. For each layer, white colonies were picked and transferred to individual wells in a deep 96-well plate containing 500 μL of LB medium, 10% glycerol and 100 μg mL^-1^ ampicillin, and were grown overnight at 37°C. Ninety-six clones per layer were shipped in dry ice to Beckman Coulter Genomics (Danvers, MA, USA) for Sanger sequencing of the 16S rRNA gene on an ABI PRISM 3730xl DNA analyzer (Applied Biosystems, Foster City, CA, USA). Clones with the *mcrA* gene insert were verified by PCR using plasmid specific M13 primers, purified using the PureLink Quick Plasmid Miniprep kit (#K210010) (Life Technologies, Carlsbad, CA) and submitted to ELIM Biopharmaceuticals, Inc. (Hayward, CA, USA) for Sanger sequencing on an ABI PRISM 3720xl DNA analyzer.

### Nucleotide sequence data and classification

Full length Sanger reads were obtained through Beckman Coulter Genomics single pass standard sequencing read service using the primers T3 (5’ -ATTAACCCTCACTAAAGGGA- 3’), T7 (5’ -TAATACGACTCACTATAGGG- 3’) and 515 (5’ -GTGCCAGCAGCCGCGGTAA- 3’) for the 16S sequences. The three reads per sample were quality-filtered, trimmed and assembled to full 16S sequences (~1.5 kb) using the software Geneious (V. 7.1.6; Biomatters Ltd., Auckland, NZ). Assemblies containing less than 3 reads were discarded. Full 16S sequences were aligned with the *E*. *coli* strain K-12 (U00096) as a reference using the SILVA Incremental Aligner (SINA) (v1.2.11) program provided by the SILVA ribosomal RNA project; http://www.arb-silva.de/aligner/) as described in Pruesse et al. [38]. Columns in the alignments with common gaps were removed using the filter.seqs command in the open-source software MOTHUR (http://www.mothur.org/) [39]. Chimeras were identified using the software Mallard (v. 1.02) with a cut-off line set to 99.9%, as described in Ashelford et al. [40]. The nucleotide sequence of outliers (samples with deviation from expectation (DE) values above the set cut-off line in Mallard) were queried manually against the NCBI non-redundant nucleotide sequence database using the BLASTN program (http://blast.ncbi.nlm.nih.gov/Blast.cgi) [41] to further assess if these were real chimeras, and were discarded if chimeric breakpoints in sequences were evident in BLAST results. After quality control, we ended with a total of 317 16S sequences, with 71, 76, 85, 85 for the top, middle, bottom and sediment layer, respectively. Single pass standard sequencing reads for *mcrA* using the M13 F primer were obtained through ELIM Biopharmaceuticals, Inc. Sequences were quality filtered and manually trimmed using CodonCode Aligner (v4.0.4; CodonCode Corp., Centerville, MA) and aligned using Clustal X 2.1 [42], a function in the software MEGA6 [43].

The search and classify tool in SINA was used for taxonomic assignment of the 16S sequences based on the last common ancestor method. Representative sequences for each phylum were further queried against the NCBI nucleotide database using the BLASTN program. Sequences identified as belonging to the genus *Salinibacter* were further analyzed in MOTHUR [39]. The cluster command was used to identify operational taxonomic units (OTUs) with a 0.01 cutoff using the average neighbor algorithm.

All sequences determined in this study were deposited in the NCBI database under GenBank accession numbers KU308828—KU309144 for 16S rRNA sequences, and KU514395-KU514406 for *mcrA* sequences.

## Results

Water in the shallow pools was uniformly 30°C at the time of measurement. The results are shown in Table 1 as the average and standard deviation of three replicate samples at two nearby sites. Although the two samples only differ only slightly in salinity, the oxygen levels differ by a factor of ~2, but with large variation between replicates. Methane concentration is high in both samples indicating a source of methane in the salt flat. Carbon dioxide and methane are distinct between the two samples (no overlap of the error bars) and they co-vary.

**Table 1:** Salinity and pH of water samples, and gas composition of released bubbles from the sabkha subsurface.

Salinity, ppt	CH_4_, ppm	δ^13^CH_4_, ‰	pH	CO_2_, %	O_2_, %	N_2_, %
320	3442 ± 343	-38.46 ± 1.25	7.67	0.65 ± 0.02	0.96 ± 0.07	97.45 ± 0.40
340	2257 ± 154	-41.14 ± 0.27	7.67	0.22 ± 0.05	1.73 ± 0.98	96.61 ± 1.07

The presence of methanogenic microorganisms at this site was confirmed through the amplification of the *mcrA* gene (~470 bp) from the bottom and sediment layers; the gene was not amplified from the top and middle layers. Twelve sequences obtained from these samples yielded close matches (>91% nucleotide identity scores) to methanogenic archaea: the extremely halophilic *Methanohalobium evestigatum* (CP002069) [44], the moderately halophilic *Methanolobus vulcani* (U22245) and *Methanohalophilus portucalensis* (AB908273) [45,46] based on BLAST analysis.

**Fig 7:**
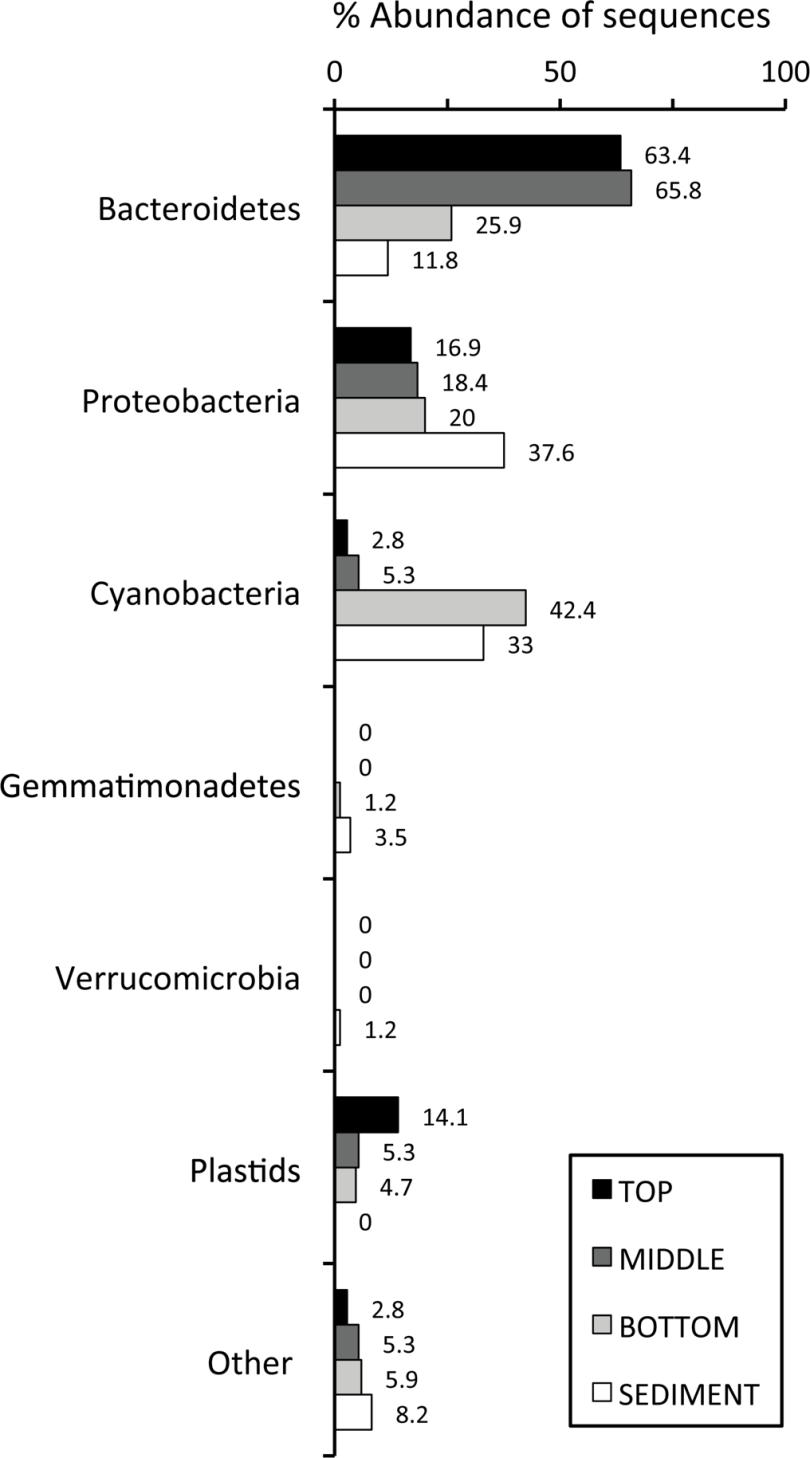
Abundance of bacterial 16S rRNA genes for each phylum for the top, middle, bottom and sediment layers of the mat. All sequences were classified using SINA program, with the exception of the plastids category, which was identified by BLASTN. A plastids category was included along with the different phyla, to indicate the presence of phototrophic eukaryotes.

Further analysis of the salt crust revealed the presence of a diverse microbial community with unusual layering with primary productivity based on photosynthesis. Based on the distribution of identified environmental sequences, the top and middle layers of the endoevaporitic mat were dominated by the phylum *Bacteroidetes* (~63.4%), with a gradual decrease in abundance with increasing depth (Fig 7). Sequences most similar to those from the genus *Salinibacter*, an extreme halophile that thrives at salinity levels of 20–30% [47], was the dominant representative in the top three layers.

Sequences for mild to moderate halophiles of the genera *Gracilimonas*, *Marivirga* (0.5–10%) and *Owenweeksia* (1–7.5%) were only identified in the sediment layer (Table 2) [48–50]. In contrast, cyanobacterial sequences were only found in low abundances in the top and middle layers (2.8 and 5.3%, respectively), and higher abundance in the bottom and sediment layers at 42.4 and 33%, respectively (Fig 7). Representative cyanobacterial sequences identified were most similar to the unicellular, halotolerant, coccoid *Cyanothece* sp., *Euhalothece* sp. and *Dactylococcopsis salina*.

**Table 2:** Select identities of closest cultured matches based on longest sequences for the phyla identified in each layer, using the NCBI BLASTN program. Proteobacteria representatives are additionally identified as belonging to the class *Alphaproteobacteria* (A), *Deltaproteobacteria* (D), or *Gammaproteobacteria* (G). The sequence similarity is indicated by ID% with similarities of 99% marked with bold type.

Layer	Phylum	Most related sequences	ID%	Accession #
**Top**				
	*Bacteroidetes*	*Salinibacter sp*.	97	AY987850
		*Salinibacter luteus*	**99**	NR_117935
		*Salinibacter iranicus*	**99**	NR_117934
	*Proteobacteria*	*Arhodomonas recens* (G)	95	NR_118045
		*Thioalkalivibrio sulfidophilus* (G)	92	NR_074692
		*Limimonas halophila* (A)	**99**	NR_109490
		*Azospirillum sp*. (A)	90	EU264075
		*Erythrobacter litoralis* (A)	98	NR_074349
	*Cyanobacteria*	*Cyanothece sp*. *104*	**99**	DQ243687
	Plastids	*Dunaliella tertiolecta*	98	JQ039090
**Middle**				
	*Bacteroidetes*	*Salinibacter sp*.	98	AY987850
		*Salinibacter iranicus*	**99**	NR_117934
	*Proteobacteria*	*Thiohalorhabdus denitrificans* (G)	93	EU374712
		*Arhodomonas recens* (G)	94	NR_118045
		*Proteobacterium FA350* (D)	87	KM034744
		*Aquisalimonas sp*. (G)	98	KC577145
		*Limimonas halophila* (A)	**99**	NR_109490
	*Cyanobacteria*	*Cyanothece sp*. *115*	**99**	DQ243690
		*Euhalothece sp*.	97	AJ000710
	Plastids	*Crustomastix stigmatica*	84	FN563093
**Bottom**				
	*Bacteroidetes*	*Salinibacter ruber*	96	CP000159
		*Salinibacter sp*.	96	KF569484
	*Proteobacteria*	*Limimonas halophila* (A)	**99**	NR_109490
		*Arhodomonas recens* (G)	94	NR_118045
		*Proteobacterium FA350* (D)	85	KM034744
	*Cyanobacteria*	*Euhalothece sp*.	96	AJ000713
	*Gemmatimonadetes*	*Rubrobacter xylanophilus*	83	NR_074552
	Plastids	*Crustomastix stigmatica*	84	FN563093
**Sediment**				
	*Bacteroidetes*	*Salinibacter sp*.	96	KF569484
		*Gracilimonas sp*.	96	KJ206435
		*Salinibacter ruber*	95	AF323502
		*Salinibacter iranicus*	96	NR_117934
		*Marivirga tractuosa*	98	NR_074493
	*Proteobacteria*	*Methylohalomonas lacus* (G)	95	NR_043973
		*Halomonas gudaonensis* (G)	**99**	NR_043807
		*Thioalkalivibrio sulfidophilus* (G)	91	NR_074692
		*Halospina denitrificans* (G)	97	NR_043525
		*Erythrobacter litoralis* (A)	98	NR_074349
		*Aquisalimonas sp*. (G)	**99**	KC577145
	*Cyanobacteria*	*Cyanothece sp*. *104*	**99**	DQ243687
		*Euhalothece sp*.	96	AJ000713
		*Dactylococcopsis salina*	**99**	NR_102465
	*Gemmatimonadetes*	*Thermaerobacter marianensis*	83	CP002344
	*Verrucomicrobia*	*Coraliomargarita akajimensis*	95	NR_074901

The taxonomic distribution of members of the *Bacteroidetes* and *Cyanobacteria* phyla along the vertical profile are consistent with the presence of a salinity gradient in this sabkha, where the top layer of the endoevaporitic mat has a high salinity, and the lower layer is influenced by a lower salinity due to the presence of fresh groundwater.

Sequences from the phylum *Proteobacteria* were present in all four layers, but were almost twice as abundant in the sediment layer, which was mostly composed of fine sand. Based on genus-level identities provided by the SINA analysis, this phylum was the most diverse in our clone libraries in terms of richness. Within these sequences, representatives of the *Alphaproteobacteria*, *Gammaproteobacteria*, and *Deltaproteobacteria* classes were identified (Table 2). Sequences with low-identity to the *Gemmatimonadetes* and sequences most similar to *Verrucomicrobia* were less common and only present in the lower two layers. Sequences with unknown identity were categorized as “other” and varied from 2.8 to 8.2% in abundance of total sequences.

Plastid sequences belonging to phototrophic eukaryotes were also identified and placed in a “plastids” category. These were present in the top (14.1%), middle (5.3%) and bottom (4.7%) layers (Fig 7). The closest identities to these plastids were for the unicellular, flagellated marine Chlorophytes *Dunaliella tertiolecta* and *Crustomastix stigmatica* (Table 2). *Dunaliella* was also observed through light microscopy in fresh mat samples. Due to their motility via flagella, these phototrophic eukaryotes may not only be able to move between layers, but also populate the water below the endoevaporitic crusts.

After additional curation to remove shorter sequences, 110 of the clones identified by SINA as most similar to *Salinibacter* were further examined using an OTU-based approach. For the top, middle, bottom, and sediment samples, there were 40, 45, 22 and 3 *Salinibacter* sequences, respectively. At 0.01 cutoff (99% similarity), 11 distinct OTUs were identified (Table 3). The largest OTU contained 62 sequences (56.4%), and four OTUs contained only a single sequence. Although the present survey only allows for qualitative comparisons of the depth distribution of these OTUs, there appeared to be a by-sampling partition for OTUs 1, 2 and 5 (mainly in the top and middle samples), and OTUs 3 and 4 (mainly found in the bottom sample). OTU 6 appeared to be cosmopolitan with representation in all samples, but with only 5 sequences total, this is not definitive (Table 3).

**Table 3:** Abundance and distribution of *Salinibacter* OTUs.

	Number of clones				
OTU #	Top	Middle	Bottom	Sediment	Total
1	27	31	4	0	62
2	3	6	1	0	10
3	0	2	7	1	10
4	1	1	7	0	9
5	4	4	0	0	8
6	1	1	2	1	5
7	1	0	1	0	2
8	0	0	0	1	1
9	1	0	0	0	1
10	1	0	0	0	1
11	1	0	0	0	1
Total	40	45	22	3	110

A phylogenetic analysis of representative sequences from the OTUs with multiple sequences supported the use of a 0.01 cutoff for the clustering, but did not reveal any divergence patterns consistent with the location of the OTU within the sabkha (not shown).

## Discussion

From the physical setting of the salt flat we expect that light levels and salinity should systematically decrease with depth. The case of oxygen is more complex, the deep ground water contains oxygen, and oxygen is present in the atmosphere at the surface. However, microbial processes in the mat consume oxygen and can produce anoxic layers. Hence, while oxygen levels may decrease with depth from the surface, layers deep enough to be in the unaltered groundwater will have high oxygen content. The two salty samples (320 and 340 ppt) in Table 1 represent shallow subsurface solutions that have interacted with the salt crust and have been exposed to some extent to the ambient atmosphere. The high methane and carbon dioxide levels argue against the oxygen being preserved from the ground water; there is no evidence of vigorous mixing of ground water with material in the biological layers.

The sampling method was inadequate to directly characterize the salinity profile below the salt crust with depth in a systematic way and we make no attempt to assign a depth difference to these samples. Nor can we exclude the possibility that our samples represent material from a range of depths mixed together by the creation of the sampling pond, which would imply that the differences in salinity and oxygen are not significant. The salinity in both sampling pools indicated nearly saturated conditions (solubility of NaCl in water at 30°C is 360 ppt). In contrast, groundwater that had been pumped into a nearby holding tank for irrigation use had a salinity of 17 ppt in agreement with published data [16] indicating, as mentioned above, the groundwater has low salinity.

The methane content differs between the two samples, increasing to 3442 ppm for the lower salinity, lower oxygen, higher carbon dioxide sample. It seems plausible that methane content would be higher in the lower salinity layers with lower oxygen levels. However, we note that we cannot rule out migration of methane gas through the system against, or with, the flow of water. Clearly further measurements are required to understand the depth profile.

The presence of methanogens at this site was strongly suggested by the amplification of the *mcrA* gene, unique to methanogens, from the bottom two layers of the mat and high sequence identities to three phylogenetic groups of methanogenic archaea. The presence of methanogens was also confirmed by the detection of methane (0.3% v/v) in bubbles collected from this site, with δ^13^C values comparable to those reported in endoevaporitic mats [4,30,51]. The unusually enriched δ^13^C values (-41 and -38‰) are higher than the traditional δ^13^C values expected for biogenic methane (~<-50‰) [4,51–53], but may be explained by lower isotopic fractionation in systems where substrate is limited, such as in high-salinity endoevaporitic mats [4,30,51]. Methane oxidation may also result in more enriched δ^13^C values, but methanotrophs are thought to be mostly absent in hypersaline environments [54]. However, our 16S sequences did include one representative with similarity to the methanotrophic *Methylohalomonas lacus* in the sediment layer (Table 2).

The significance of the methane is twofold. First, it represents a source of methane in a desert environment on Earth contributing to the global inventory of methane sources. Secondly, methane production in this sabkha provides one possible model for methane production on Mars, as discussed below.

Studies of orbital images over the past 40 years show no detectable change in the position of the dunes or the location of the sabkhas. This indicates that the timescale for covering and uncovering new sabhka flats is in excess of hundreds of years. Based on satellite images and GPS ground observations over several years, Lorenz and Radebaugh [55] computed a net motion of ~0.1 m yr^-1^ advance for the large dunes of the Liwa Oasis. The sabhka studied here was about 500 m in diameter (Fig 2) suggesting a lifetime against burial by the large dunes of 5000 years. It may be possible to discover previously buried sabkha in this area exposed by recent movement of the dunes and thus investigate the preserved remains of past mat systems.

An important contrast between this environment and previous studies of salt ponds and coastal sabkhas is the salinity gradient. In most hypersaline environments in deserts or coastal sites the salt arrives with the water supply from above the surface and accumulates as the water evaporates. In the Liwa Oasis sabkha studied here, the water flow is in the subsurface; there is very little surface flow. This creates an unusual layered structure with saturated salt layers on top and relatively freshwater layers below. The structure of the microbial mat (Fig 5) is the clearest indication we have of this vertical structuring due to light, salinity, and oxygen gradients. Clone libraries constructed from four depths within the sabkha crust showed changes in taxonomic composition consistent with decreasing salinity with depth (e.g., reduced total and proportional abundances of *Salinibacter* sequences), but further research is required to understand the role this gradient has on structuring the microbial community. This gradient is shown schematically in Fig 6.

The interdune sabkhas in Liwa Oasis are of particular interest as a Mars analog. The salt crust is a relic deposited by previous evaporation. Although widespread, the salt crust is not easily visible by remote sensing because it is mixed with sand. Similar relic salt flats may exist on Mars today, remnants from a wetter period in the past buried by the present shifting sands. The sabkha studied here suggests an alternative way these salt deposits could support life. If there was a source of deep ground water flowing upward from below, a near-surface habitable layer might be formed protected from the low pressure, arid surface conditions. The results from the Liwa Oasis suggest a microbial model for such a habitat and for methane production at such a site. Light can penetrate the salt crust providing energy for photosynthesis but the crust provides a barrier to loss of water into the dry atmosphere due both to physically blocking exchange between the water and the atmosphere and by lowering the vapor pressure of water due to solution. This reduction of water loss by the salt layer operates in the Liwa Oasis as it would on Mars.

In the Liwa Oasis, methane is being released from the salt community continuously as the salt pan is fractured in many places. This would not be the case on Mars as the pan would have to be tightly sealed to preserve the liquid water habitat below. On Mars methane would build up below the salt pan until the gas pressure exceeded the pressure holding the salt pan down, the vapor pressure of water, assuming it is cold, would barely exceed the ambient pressure and hence alone would not suffice to break the surface. The pan would then crack with a sudden release of gas. Rapid evaporation and flash freezing of the outflowing water on the surface would quickly reseal the crack with ice, which would slowly sublime leaving behind sediment and salt. The cycle might repeat on timescale of years or even decades. Such a system could conceivably explain large outbursts of methane on Mars as has been reported [56]. However it does not provide an explanation for the subsequent rapid loss of the methane plumes also reported [56]. This rapid loss is inconsistent with known photochemical processes on Mars [57].

A salt pan system such as studied here can prolong habitability for photosynthetic surface biology when only ground water is available and surface conditions are unsuitable for stable liquid water. Another important aspect of this system is the preservation of biomarkers, including methane and other evidence for life in the salt after it dries out. Such a near surface system could be accessed by surface rovers. If an inverted salt mat system fed by groundwater existed on Mars in the past but is no longer operative it may still be of considerable astrobiology interest. It has been suggested [2] that salt deposits can preserve evidence of past life. As the flow of water to the inverted mat ceased, the active microbial system would be encased in salt and preserved over geological time, relatively protected from radiation and oxidation damage.

## References

[1] Edwards HGM, Mohsin MA, Sadooni FN, Hassan NFN, Munshi T. Life in the sabkha: Raman spectroscopy of halotrophic extremophiles of relevance to planetary exploration. Anal. Bioanal. Chem. 2006;385: 46–56. 16607492 10.1007/s00216-006-0396-3

[2] Rothschild LJ. Earth analogs for Martian life. Microbes in evaporites a new model system for life on Mars. Icarus 1990;88: 246–60. 11538366 10.1016/0019-1035(90)90188-f

[3] Rothschild LJ, Giver LJ, White MR, Mancinelli RL. Metabolic activity of microorganisms in evaporates. J. Phycol. 1994;30: 431–438. 11539827 10.1111/j.0022-3646.1994.00431.x

[4] Tazaz AM, Bebout BM, Kelley CA, Poole J, Chanton JP. Redefining the isotopic boundaries of biogenic methane: Methane from endoevaporites. Icarus 2013;224: 268–275.

[5] Davila AF, Duport LG, Melchiorri R, Janchen J, Valea S, de Los Rios A, et al Hygroscopic salts and the potential for life on Mars. Astrobiology 2010;10: 617–628. 10.1089/ast.2009.0421 20735252

[6] Wierzchos J, Ascaso C, McKay CP. Endolithic cyanobacteria in halite rocks from the hyperarid core of the Atacama Desert. Astrobiology 2006;6: 415–422. 16805697 10.1089/ast.2006.6.415

[7] Douglas S. Microbial biosignatures in evaporite deposits: Evidence from Death Valley, California. Plant. Space Sci. 2004;52: 223–227.

[8] Douglas S, Yang H. Mineral biosignatures in evaporites: Presence of rosickyite in an endoevaporitic microbial community from Death Valley, California. Geology 2002;30: 1075–1078.

[9] Douglas S, Abbey W, Mielke R, Conrad P, Kanik I. Textural and mineralogical biosignatures in an unusual microbialite from Death Valley, California. Icarus 2008;193: 620–636.

[10] Barbieri R, Stivaletta N, Marinangeli L, Ori GG. Microbial signatures in sabkha evaporite deposits of Chott el Gharsa (Tunisia) and their astrobiological implications. Planet. Space Sci. 2006;54: 726–36.

[11] Al-Farraj A. An evolutionary model for sabkha development on the north coast of the UAE. J. Arid Environ. 2005;63: 740–55.

[12] Evans G. Coastal and nearshore sedimentation: A comparison of clastic and carbonate deposition. Proceedings of the Geologists' Association. 1970;81: 493–508.

[13] Shinn EA, Robbin DM. Mechanical and chemical compaction in fine-grained shallow-water limestones. J. Sediment. Petro. 1953;53: 595–618.

[14] El-Sayed MI. The nature and possible origin of mega-dunes in Liwa Ar Rub' Al Khali UAE. Sediment. Geol. 2000;134: 305–330.

[15] El-Sayed MI. Sedimentological characteristics and morphology of the aeolian sand dunes in the eastern part of the UAE a case study from Ar Rub' Al Khali. Sediment. Geol. 1999;123: 219–238.

[16] Wood WW, Imes JL. Dating of Holocene Ground-water recharge in western part of Abu Dhabi (United Arab Emirates): Constraints on global climate-change models. Developments Water Sci. 200350: 379–385.

[17] Ojha L, Wilhelm MB, Murchie SL, McEwen AS, Wray JJ, Hanley J, et al Spectral evidence for hydrated salts in recurring slope lineae on Mars. Nature Geosci. 2015;8: 829–832.

[18] Davila AF, Gross C, Marzo GA, Fairén AG, Kneissl T, McKay CP, et al A large sedimentary basin in the Terra Sirenum region of the southern highlands of Mars. Icarus 2011;212: 579–589.

[19] Osterloo MM, Hamilton VE, Bandfield JL, Glotch TD, Baldridge AM, Christensen PR, et al Chloride-bearing materials in the southern highlands of Mars. Science 2008;319: 1651–1654. 10.1126/science.1150690 18356522

[20] Osterloo MM, Anderson FS, Hamilton VE, Hynek BM. Geologic context of proposed chloride-bearing materials on Mars. J. Geophys. Res. 2010

[21] Al-Katheeri ES, Howari FM, Murad AA. Hydrogeochemistry and pollution assessment of quaternary–tertiary aquifer in the Liwa area United Arab Emirates. Environ. Earth Sci. 2009;59: 581–592.

[22] Wood WW. Source of paleo-groundwater in the Emirate of Abu Dhabi, United Arab Emirates: evidence from unusual oxygen and deuterium isotope data. Hydrogeo. J. 2011;19: 155–161.

[23] Wood WW, Imes JL. How wet is wet? Constraints on late Quaternary climate in the southern Arabian Peninsula. J. Hydrol. 1995;164: 263–268

[24] Wood WW, Sanford WE, Al Habschi ARS. The source of solutes in the coastal sabkha of Abu Dhabi. Bull. Geol. Soc. Am. 2002;114: 259–268.

[25] Wood WW, Rizk ZS, Alsharhan AS. Timing of recharge, and the origin, evolution and distribution of solutes in a hyperarid aquifer system. Developments Water Sci. 2003;50: 295–312.

[26] Wood WW, Rizk ZS, Alsharhan AS. Timing of recharge, and the origin, evolution, and distribution of solutes in a hyperarid aquifer system In: Alsharhan AS, Wood WW, editors. Water Resources Perspectives: Evaluation, Management and Policy. Amsterdam: Elsevier; 2003 pp. 295–312.

[27] Böer B. An introduction to the climate of the United Arab Emirates. J. Arid Environ. 1997;35: 3–16.

[28] Glennie KW. The desert of southeast Arabia: a product of Quaternary climate change In: Alsharhan A, Glennie K, Whittle G, Kendall C, editors. Quaternary Deserts and Climatic Change. Rotterdam: Balkema; 1998 pp 279–291.

[29] Bebout BM, Hoehler TM, Thamdrup B, Albert D, Carpenter SP, Hogan M, et al Methane production by microbial mats under low sulphate concentrations. Geobiology 2004;2: 87–96.

[30] Kelley CA, Poole JA, Tazaz AM, Chanton JP, Bebout BM. Substrate limitation for methanogenesis in hypersaline environments. Astrobiology 2012;12: 89–97. 10.1089/ast.2011.0703 22248383

[31] Oren A. Salinibacter: an extremely halophilic bacterium with archaeal properties FEMS Microbiol. Lett. 2013;342: 1–9. 10.1111/1574-6968.12094 23373661

[32] Webster CR, Mahaffy PR, Atreya SK, Flesch GJ, Mischna MA, Conrad PG, et al Mars methane detection and variability at Gale crater. Science 2015;347: 415–417. 10.1126/science.1261713 25515120

[33] Rice AL, Gotoh AA, Ajie HO, Tyler SC. High-precision continuous-flow measurement of δ^13^C and δD of atmospheric CH_4_. Anal. Chem. 2001;73: 4104–4110. 11569798 10.1021/ac0155106

[34] Turner S, Pryer KM, Miao VPW, Palmer JD. Investigating deep phylogenetic relationships among cyanobacteria and plastids by small subunit rRNA sequence analysis. J. Eukaryotic Microbio. 1999;46: 327–338. 10.1111/j.1550-7408.1999.tb04612.x 10461381

[35] Lane DJ. 16S/23S rRNA sequencing In: Nucleic acid techniques in bacterial systematics. Stackebrandt E., Goodfellow M, editors. New York: John Wiley and Sons; 1991 pp. 115–175.

[36] Luton PE, Wayne JM, Sharp RJ, Riley PW. The *mcrA* gene as an alternative to 16S rRNA in the phylogenetic analysis of methanogen populations in landfill. Microbio. 2002;148: 3521–3530. 10.1099/00221287-148-11-3521 12427943

[37] Garcia-Maldonado JQ, Bebout BM, Everroad RC, López-Cortés A. Evidence of novel phylogenetic lineages of methanogenic archaea from hypersaline microbial mats. Microbial. Eco. 2014;69: 106–117. 10.1007/s00248-014-0473-7 25108574

[38] Pruesse E, Peplies J, Glockner FO. SINA: accurate high-throughput multiple sequence alignment of ribosomal RNA genes. Bioinformatics 2012;28: 1823–1829. 10.1093/bioinformatics/bts252 22556368 PMC3389763

[39] Schloss PD, Westcott SL, Ryabin T, Hall JR, Hartmann M, Hollister EB, et al Introducing mothur: open-source, platform-independent, community-supported software for describing and comparing microbial communities. Appl. Environ. Microbiol. 2009;75: 7537–7541. 10.1128/AEM.01541-09 19801464 PMC2786419

[40] Ashelford KE, Chuzhanova NA, Fry JC, Jones AJ, Weightman AJ. New screening software shows that most recent large 16S rRNA gene clone libraries contain chimeras. App. Environ. Microbio. 2006;72: 5734–5741. 10.1128/AEM.00556-06 PMC1563593 16957188

[41] Zhang Z, Schwartz S, Wagner L, Miller W. A greedy algorithm for aligning DNA sequences. J. Comput. Bio. 2000;7: 203–214. 10890397 10.1089/10665270050081478

[42] Larkin MA, Blackshields G, Brown NP, Chenna R, McGettigan PA, McWilliam H, et al Clustal W and Clustal X version 2.0. Bioinformatics 2007;23: 2947–2948. 17846036 10.1093/bioinformatics/btm404

[43] Tamura K, Stecher G, Peterson D, Filipski A, Kumar S. MEGA6: molecular evolutionary genetics analysis version 6.0. Molec. Bio. Evol. 2013;30: 2725–2729. 24132122 10.1093/molbev/mst197 PMC3840312

[44] Zhilina TN, Zavarzin GA. Extremely halophilic, methylotrophic, anaerobic bacteria. FEMS Microbio. Rev. 1990;7: 315–321.

[45] Antony CP, Murrell JC, Shouche YS. Molecular diversity of methanogens and identification of Methanolobus sp. as active methylotrophic Archaea in Lonar crater lake sediments. FEMS Microbio. Eco. 2012;81: 43–51. 10.1111/j.1574-6941.2011.01274.x 22150151

[46] Wilharm T, Zhilina TN, Hummel P. DNA-DNA Hybridization of Methylotrophic Halophilic Methanogenic Bacteria and Transfer of Methanococcus halophilusvp to the Genus Methanohalophilus as Methanohalophilus halophilus comb. nov. Int. J. Systematic Bact. 1991;41: 558–562.

[47] Oren A. Anaerobic degradation of organic compounds at high salt concentrations. Antonie van Leeuwenhoek 1988;54: 267–277. 3048206 10.1007/BF00443585

[48] Pagani I, Chertkov O, Lapidus A, Lucas S, Del Rio TG, Tice H, et al Complete genome sequence of *Marivirga tractuosa*type strain (H-43). Standards Genomic Sci. 2011;4: 154–162. 10.4056/sigs.1623941 PMC3111994 21677852

[49] Riedel T, Held B, Nolan M, Lucas S, Lapidus A, Tice H. et al Genome sequence of the orange-pigmented seawater bacterium *Owenweeksia hongkongensis* type strain (UST20020801^T^). Standards Genomic Sci. 2012;7: 120–130. 10.4056/sigs.3296896 PMC3570807 23450211

[50] Wang YX, Li YP, Liu JH, Xiao W, Lai YH, Li ZY, et al Gracilimonas mengyeensis sp. nov., a moderately halophilic bacterium isolated from a salt mine in Yunnan, south-western China. Int. J. Syst. Evol. Microbiol. 2013;63, 3989–3993. 10.1099/ijs.0.052043-0 23710054

[51] Kelley CA, Nicholson BE, Beaudoin CS, Detweiler AM, Bebout BM. Trimethylamine and organic matter additions reverse substrate limitation effects on the δ^13^C values of methane produced in hypersaline microbial mats. App. Environ. Microbio. 2014;80: 7316–7323. 10.1128/AEM.02641-14 PMC4249174 25239903

[52] Sherwood-Lollar B, Lacrampe-Couloume G, Slater GF, Ward J, Moser DP, Gihring TM, et al Unravelling abiogenic and biogenic sources of methane in the Earth's deep subsurface. Chem. Geo. 2006;226: 328–339.

[53] Whiticar MJ. Carbon and hydrogen isotope systematics of bacterial formation and oxidation of methane Chem. Geo. 1999;161: 291–314.

[54] Conrad R, Frenzel P, Cohen Y. Methane emission from hypersaline microbial mats: Lack of aerobic methane oxidation activity. FEMS Microbiol. Ecol. 1995;16: 297–306.

[55] Lorenz RD, Radebaugh J. Giant Linear Dunes as the Formation Pathway to Megabarchan Chains: Titan and the Rub 'Al Khali. Fourth International Planetary Dunes Workshop, 2015; abstract 8003, www.hou.usra.edu/meetings/dunes2015/pdf/8003.pdf

[56] Mumma MJ, Villanueva GL, Novak RE, Hewagama T, Bonev BP, DiSanti MA, et al Strong release of methane on Mars in northern summer 2003. Science, 2009;323: 1041–1045. 10.1126/science.1165243 19150811

[57] Zahnle K, Freedman RS, Catling DC. Is there methane on Mars? Icarus 2011;212: 493–503.

